# Association Between *aquaporin-1* and Endurance Performance: A Systematic Review

**DOI:** 10.1186/s40798-019-0213-0

**Published:** 2019-09-05

**Authors:** Miguel A. Rivera, Thomas D. Fahey

**Affiliations:** 1 Department of Physical Medicine, Rehabilitation & Sports Medicine, School of Medicine, University of Puerto Rico, Main Building Office A204, San Juan, PR 00936 USA; 2 Department of Kinesiology, California State University, Chico, 95929–0330 CA USA

**Keywords:** Aquaporin 1, Genetics, rs1049305 variant, Distance running, Athletes

## Abstract

**Background:**

There is abundant and mounting information related to the molecular and biological structure and function of the *Aquaporin-1* (*AQP1*) gene and the AQP1-Aquaporin channel. Regulation of water flow across cell membranes is essential for supporting inter- and intracellular fluid balance, which is critical for health and exercise performance. The transmembrane water channel AQP1 is important for cardiorespiratory endurance (CE) because it influences fluid transfers in erythrocytes, endothelial, and pulmonary cells and is vital for transport of ammonium, bicarbonate, carbon dioxide, glycerol, nitric oxide, potassium ion, water, and trans-epithelial and renal water. Very recent publications suggest the association between a DNA sequence variant, rs1049305 (C > G), in the 3′-untranslated region of the *AQP1* gene and CE performance. Other reports indicate further significant associations between AQP1 channel and CE phenotypes. The purposes of this systematic review were to examine the extent of the associations between the *AQP1* rs1049305 genotype and CE exercise performance and body fluid loss in long-distance runners and AQP1 channel associations with other CE phenotypes.

**Methods:**

Data sources: A comprehensive review was conducted using PubMed, EMBASE, CINAHL, and Cochrane electronic databases. The search ranged from January 1, 1988, to December 31, 2018. Studies reported in English, French, and Spanish were considered. Eligibility criteria: The criteria for inclusion in the review were (a) case-control study; (b) unequivocal definition of cases and controls; (c) CE was defined as performance in endurance events, laboratory tests, and/or maximal oxygen consumption; (d) exclusion criteria of known causes; (e) genotyping performed by PCR or sequencing; (f) genotype frequencies reported; and (g) no deviation of genotype frequencies from Hardy-Weinberg equilibrium in the control group. Study appraisal: The systematic review included studies examining the *AQP1* gene and AQP1 channel structure and function, associations between the *AQP1* gene sequence variant rs1049305 (C > G)  and CE performance, body fluid loss in long-distance runners, and other studies reporting on the *AQP1* gene and channel CE phenotype associations. Synthesis methods: For each selected study, the following data were extracted: authors, year of publication, sample size and number of cases and controls, CE definition, exclusion criteria, inclusion criteria for cases and controls, methods used for genotyping, genotype, allele frequencies and HWE for genotype frequencies in cases and control groups, and method of *AQP1* gene and AQP1 channel analysis.

**Results:**

The initial databases search found 172 pertinent studies. Of those, 46 studies were utilized in the final synthesis of the systematic review. The most relevant findings were (a) the identification of an independent replication of the association between *AQP1* gene sequence variant rs1049305 (C > G) and CE performance; (b) the association of the rs1049305 C-allele with faster CE running performance; (c) in knockout model, using a linear regression analysis of distance run as a function of Aqp1 status (Aqp1-null vs. wild-type mice) and conditions of hypoxia (ambient [O_2_] = 16%), normoxia (21%), and hyperoxia (40%) indicated that the Aqp1 knockout ran less distance than the wild-type mice (*p* < 0.001); (d) in vitro, a reduced *AQP1* expression was associated with the presence of the rs1049305 G-allele; (e) AQP1 null humans led normal lives and were entirely unaware of any physical limitations. However, they could not support fluid homeostasis when exposed to chronic fluid overload. The limited number of studies with “adequate sample sizes” in various racial and ethnic groups precluding to perform proper in-depth statistical analysis.

**Conclusions:**

The *AQP1* gene and AQP1 channel seems to support homeostatic mechanisms, yet to be totally understood, that are auxiliary in achieving an advantage during endurance exercise. AQP1 functions are vital during exercise and have a profound influence on endurance running performance. AQP1s are underappreciated structures that play vital roles in cellular homeostasis at rest and during CE endurance running exercise. The outcome of the present systematic review provide support to the statement of hypotheses and further research endeavors on the likely influence of *AQP1* gene and AQP1 channel on CE performance. *Registration*: The protocol is not registered.

## Key Points

- The *AQP1* gene and AQP1 channel functions are vital during exercise and have profound influence on cardiorespiratory endurance performance.
- This is significant because genetic (molecular) mechanisms and their effects on cardiorespiratory endurance performance phenotypes are major areas of inquiry in the science and medicine of sport and exercise.
- Regulation of water flow across cell membranes is essential for supporting a proper fluid balance within the cells, which is a critical factor in health status and endurance performance.

## Background

The concept of “genetic mechanisms” and their effect on cardiorespiratory endurance (CE) performance phenotypes is a major area of inquiry in the science and medicine of sport and exercise. CE is the ability of the body to perform prolonged, large-muscle, dynamic exercise at moderate to high levels of intensity [1]. The best clinical and physiological criterion of CE is the maximal oxygen consumption (VO_2max_). The latter is the highest rate at which the body takes up, transports, and uses oxygen at sea level. CE and the VO_2max_ measures are high, significant, and positively correlated, and both are highly influenced by genetic mechanisms [2]. An intriguing area of research is the relationship between CE exercise performance and molecular genetic mechanisms related to water and solute transport across membranes. The body mass of humans is approximately 70% water. Regulation of water flow across cell membranes is essential for maintaining an appropriate fluid balance within the cells [3], which is a critical factor in health status and CE performance [4]. A novel finding related to water management at the molecular level occurred in 1988 with the identification of transmembrane water channels in erythrocytes [5]. That work led to a Nobel Prize in Chemistry for Peter Agre in 2003. The capacity of the erythrocytes to take up and release water make them significant in controlling body water distribution throughout the body [6]. The transmembrane water channel protein, which was originally named CHIP28, is now known as Aquaporin-1 (AQP1) [7]. There is abundant and mounting information related to the molecular and biological structure and function of the *AQP1* gene and the AQP1 channel [8].

### Aquaporin-1 Gene and Channel

Aquaporins (AQPs) are a family of transmembrane proteins divided into two subfamilies: those which transport only water, and aquaglyceroporins, which transport water and small organic compounds [9]. The AQP1 channel is the best known and most studied of the AQP family. The AQP1 channel is encoded by the *AQP1* gene, on chromosome 7, region p14 [10]. This gene extends 17 kb pairs and contains four exons and three introns [7]. The *AQP1* gene is highly polymorphic, displaying over 150 deoxyribonucleic acid (DNA) sequence variations [11]. The AQP1 channel is a complex, sophisticated, and regulated 28-kDa protein known to play several molecular functions and biological processes [7, 12, 13]. The channel shows transmembrane transporter activity for water, ammonium, bicarbonate, carbon dioxide, glycerol, nitric oxide, and renal water. It also shows channel activity for intracellular cGMP-activated cation, and potassium. Other known molecular activity is that of identical protein binding [12]. Moreover, it shows cellular response to cyclic guanosine monophosphate (cAMP), copper ion, dexamethasone stimulus, hydrogen peroxide, organic substance, mercury ions, retinoid acid, salt stress, ultraviolet (UV) light, hyper-osmosis, hypoxia, mechanical stimulus, and stress, also shows a positive regulation of angiogenesis, fibroblast proliferation, and saliva secretion. It negatively regulates cysteine-type endopeptidase activity involved in the apoptotic process. Further roles include secretion of cerebrospinal fluid and pancreatic juice, cGMP biosynthetic process, establishment or maintenance of actin cytoskeleton polarity, chondrocyte water homeostasis, lateral ventricle development, maintenance of symbiont-containing vacuole by host, and multicellular organismal water homeostasis [12].

The AQP1 channel exists as a tetramer with each subunit containing its own functionally independent pore [14, 15]. Each subunit contains six transmembrane helices packed to form a trapezoid-like structure [16]. The pore’s dumbbell-like shape consists of an extracellular area, a selectivity filter containing the constriction region, and a cytoplasmic area. The water permeability and the ion conductance do not occur through the same pathway [17], as the individual subunit pore transports water and the central pore acts as a selective ion channel that allows the flow of ions and water [18]. The water selectively of AQP1 is caused by the size exclusion effect [19, 20]. The narrow or mid-region at the channel imposes a strict restriction, so there is only one water molecule at a time allowed to pass through [19]. The flux of water is at approximately 10^9^–10^10^ molecules per second per channel, with an activation energy as low as the one associated with the self-diffusion rate in bulk water [19, 21, 22]. When coming close to the mid-region, the water molecule undergoes a rotation helping to form hydrogen bonds between its oxygen and specific amino groups [23]. This rotation and its effect is essential for proton exclusion [24]. The monomeric pores of AQP1 have strong hydrogen bonds between the protein and water molecules inside the channel; this makes the monomers less gas permeable than the central pore [25]. Recent evidence suggests that the hydrophobic central pore of AQP1 is permeable to both CO_2_ and O_2_ [25]. Regulating cation permeability of AQP1 through a cGMP-dependent signaling cascade has significant importance to the control of secretion and absorption of fluid, and regulation of cell volume in many tissues that highly express this channel [18]. AQP1 has been identified in various tissues, including red blood cells, endothelial cells, and smooth, skeletal, and cardiac muscles [26, 27]. The major aquaporin of the cardiovascular system is AQP1, which probably regulates water permeability of the heart’s capillary networks by mediating the flow of water through the endothelial layer into the blood. Very recent publications [28–30] suggest the association between a DNA sequence variant, rs1049305 (G > C), in the 3′-untranslated region (3′ UTR) of the *AQP1* gene and the CE performance level and body fluid loss in long-distance runners. Other reports [31, 32] indicate further significant associations between AQP1 and CE phenotypes. To the best of our knowledge, no in-depth reviews examining, describing, or analyzing such AQP1 associations have been published. There is consensus that individual reports do not provide sound and clear evidence as that provided by systematic reviews. Therefore, the purpose of this systematic review was to answer the question of the extent of the association between the *AQP1* rs1049305 (G > C) genotype and CE performance level and body fluid loss in long-distance runners, along with other studies reporting on the AQP1 and CE phenotypes associations. The outcome of the present systematic review could provide support to further research endeavors and statement of hypotheses on the possible influence of *AQP1* gene and channel on CE performance.

## Methods

### Eligibility criteria

The search examined papers published between January 1, 1998, and December 31, 2018. No limits were applied to searches and studies reported in English, French, and Spanish. The review included studies examining the *AQP1* gene and AQP1 channel structures and physiology potentially underlying CE, association between the *AQP1* gene sequence variant at reference single-nucleotide polymorphism cluster identification number (rs) 1049305 (C > G) and CE performance, and body fluid loss in long-distance runners and studies reporting on the *AQP1* gene and channel associations with other CE phenotypes.

### Inclusion criteria

The criteria to include in the review were (a) case-control study; (b) unequivocal definition of cases and controls; (c) CE defined as performance in endurance events (preferably running events), laboratory tests, and/or VO_2max_; (d) exclusion criteria of known causes; (e) genotyping performed by the polymerase chain reaction (PCR) or sequencing; (f) genotype frequencies reported; (g) no deviation of genotype frequencies from Hardy-Weinberg equilibrium (HWE) in the control group; and (h) *AQP1* gene and channel structures and functions studies potentially underlying CE .

### Information sources

A comprehensive review of the literature was conducted using PubMed, Excerpta Medica database (EMBASE), Cumulative Index of Nursing and Allied Health Literature (CINAHL), and Cochrane electronic databases detailing papers published between January 1, 1998, and December 31, 2018

### Search strategy

For each electronic database search, the following keywords were used Aquaporin-1, Aquaporin-1 gene structure, Aquaporin-1 channel structure + function, *AQP1*, AQP1, AQP1 + polymorphism + rs1049305, cardiorespiratory + endurance, body fluid loss, association studies, case-control, polymorphism, single-nucleotide polymorphisms (SNPs), VO_2max_, cardiovascular fitness, cardiorespiratory fitness, aerobic fitness, aerobic capacity, long-distance running, and body water regulation. To reduce the risk of missing studies, the reference lists of the retrieved articles were also examined, along with grey literature (produced at all levels of government, academics, business, and industry in print and electronic formats, but which are not controlled by commercial publishers) [33] or sources other than peer-reviewed journals. If merited, corresponding authors were contacted for additional information on published and unpublished studies. The authors performed the literature search independently. The retrieved publications were deposited in an electronic file (Dropbox: Dropbox Inc., San Francisco, CA) which was accessible to both authors. That avoided duplications and allowed each author to rapidly evaluate incoming documents. The authors first examined the article title for relevance to the topic and probable link to the inclusion criteria. If the title was not clear regarding the above, both authors reviewed the abstracts. When abstracts were relevant, the full text of the article was reviewed. Any disagreements were discussed and resolved by consensus.

### Data collection process

The authors extracted data items and entered them on an Excel spreadsheet. For each finally selected study, the following data were extracted: authors; year of publication; type of study; *AQP1* gene details; AQP1 channel details; population and number of cases and controls; CE definition; exclusion criteria; inclusion criteria for cases and controls; methods used for genotyping, genotype, and allele frequencies; and HWE for genotype frequencies in cases and control groups. If HWE was not informed, it was calculated by the authors. The search items were based on established terminology using Cochrane definitions where possible. Corresponding authors were contacted for other relevant information. Appropriate data extraction was corroborated by each investigator.

### Risk of bias in individual studies

The main risk of bias in individual studies is that genotyping methods differ between studies.

### Summary measures

Summary measures included genotype and allele frequencies in cases and controls, chi-square analysis of genotype and allele frequencies in cases and controls, odds ratios, chi-square analysis of genotype by C-allele carrier status and allele frequencies in cases and controls, *AQP1*-null vs wild-type phenotypes contrasts, performance level by genotype schemes, and absolute contrast between the C-allele frequency prevalence in different ethnic and racial groups.

### Synthesis of results

This involves judgment of study results by authors and how results conformed to the hypothesis dealing with *AQP1*/AQP1 and CE performance.

## Results

### Outcome of Scrutiny

**Fig. 1:**
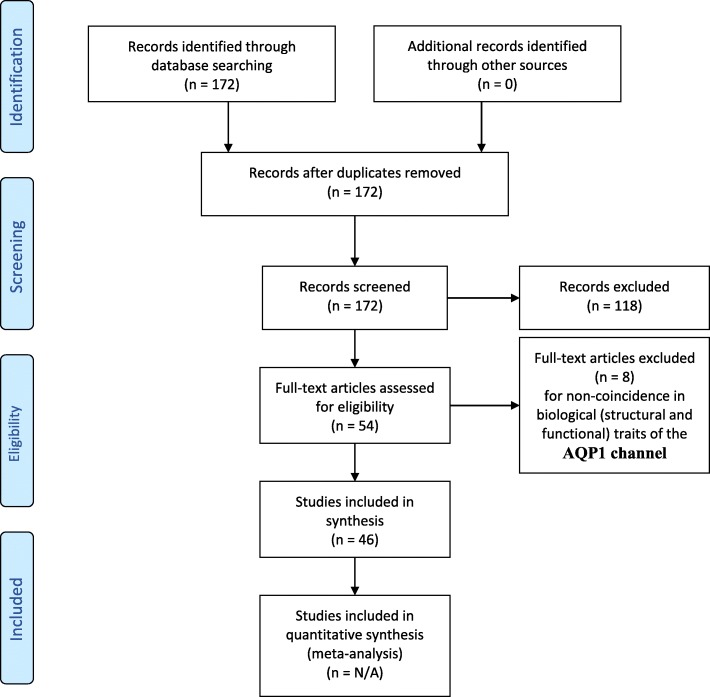
PRISMA flow chart. Details of the present search and selection process applied during the systematic review process

Figure 1 shows a flow chart of the studies’ selection process. The initial database search found 172 pertinent studies. A further screening of those 172 studies led to the exclusion of 118 publications due to marginal relevance and the retention of 54 documents for eligibility determination. That process led to the rejection of eight studies due to non-coincidence in biological (structural and functional) traits of the AQP1 channel. The final outcome yielded 46 studies that were utilized in the synthesis of the present systematic review.

### *AQP1* Case–Control and CE Performance

In humans (see Table 1), the first line of evidence to support the hypothesis of association between the *AQP1* gene and CE performance was provided by Martinez et al. [28]. That report was an observation study using a genetic epidemiology model in a case-control design. They examined the association between a DNA sequence variant, rs1049305 (C > G), in the 3′ UTR of the *AQP1* gene and CE performance level in male and female Hispanic marathon runners (*n* = 784). Cases (fast runners; *n* = 396; men = 225; women = 171) were finishers in the top third percentile for their age and sex, while controls (slow runners; *n* = 388; men = 221; women = 167) finished in the lowest third percentile. The reported genotype frequencies were in HWE (*X*^2^, *p* ≥ 0.05) and were not significantly (*X*^2^, *p* ≥ 0.05) different between sexes. Given there were similar genotypic frequency distributions in men and women, for both cases (fast runners) and controls (slow runners), the data for both sexes were pooled. Chi-square test on the pooled data revealed a significant (*X*^2^ = 6.94, *p* = 0.03) difference in the genotype prevalence distribution between the cases (fast runners) and controls (slow runners).

**Table 1:** Summary of studies on the association of AQP1gene rs1049305 (C > G) variant in the 3′ untranslated region and endurance performance in humans

Source (ref no.)	Study design	Purpose	Exclusion criteria	Subjects	Study sample		DNA source/genotyping method	Genotype* frequencies		Allele frequencies		Main finding	Odds ratio	95% confidence interval	*p* value
28	Observation during a marathon run, using a genetic epidemiology association model with a case-controls design	Association between a single-nucleotide polymorphism (SNP), restriction site (rs) 1049305 (C > G) in the 3′ UTR of the AQP1 gene and endurance performance level	Known history of cardiovascular, respiratory, arthritis, metabolic disease or on medication. Incident that negatively affected performance during the race (e.g., significant injury)	*N* = 784. Adult Hispanic marathon runners, biologically unrelated recruited between 2004 and 2008. All were informed and consented to be part of the study. There were no replications	Cases (*n* = 396) Finishers in the top third percentile of respective age and gender	Controls (*n* = 388) Finishers in the lowest third percentile of respective age and gender	Peripheral blood leucocytes tetra-primer Amplification Refractory Mutation System PCR procedure followed by gel electrophoresis	Cases *n* = 396 CC = 59 (0.15) GC = 167 (0.42 GG = 170 (0.43)	Controls *n* = 388 CC = 39 (0.10) GC = 151 (0.39) GG = 198 (0.51)	Cases *n* (%) C = 285 (0.36) G = 507 (0.64)	Controls *n* (%) C = 229 (0.30) G = 547 (0.70)	Fifty- seven percent (57%) of the cases (fast runners) were carriers of the C-allele versus 49% for the controls (slow runners)	1.35	1.08–1.67	≤ 0.005
										*X*^2^ = 7.56, df = 1, *p* = 0.005					
								Hardy-Weinberg equilibrium							
								*X*^2^ = 2.84, df = 2, *p* = 0.09	*X*^2^ = 1.62, df = 2, *p* = 0.21						
								*X^2^ = 6.94, df = 2, *p* = 0.03							
29	Observation during a 10-km run, using a Genetic Epidemiology association model with allele carrier vs. no-carrier design	Explored the association between a SNP, rs 1049305 (C > G) in the 3′ UTR region of the AQP1 gene and acute body fluid loss as well as endurance performance	Same as Martinez et al [28]	*N* = 91 Adult Hispanic males, recreational long-distance runners, biologically unrelated, were informed and consented to be part of the study during the Maratón Pacifico 10-km run	C-allele carriers, *n* = 50	C-allele non-carriers, *n* = 51	Peripheral blood leucocytes tetra-primer Amplification Refractory Mutation System PCR procedure followed by gel electrophoresis	CC *n* (%) = 10 (0.11) CG *n* (%) = 40 (0.44) GG *n* (%) = 41 (0.45)				The unrelated *t* test revealed that the C-allele carriers ran faster (approximately 16.12 km/h) than C-allele non-carriers (approximately 13.9 km/h) during a 10-km race.			
								Hardy-Weinberg equilibrium *X*^2^ = 2.84, df = 2, *p* = 0.09							
								Genotype by C-allele status				CC or CG: mean = 37 ± 2 min GG: mean = 43 ± 3 min		35–39 min 40–46 min	≤ 0.05
								Carriers CC+CG *n* = 50	Non-carriers GG *n* = 41						
30	Observation during an Ironman event, using a Genetic Epidemiology association model with allele carrier vs. no-carrier design	Tested the association of the rs1049305 (C > G) variant within the 3′ UTR of the AQP gene, with changes in body weight, post-race serum sodium concentration and performance in Ironman triathletes	No major health concerns	*N* = 476 of 504, consenting South African, Caucasian male, triathletes who completed either the 2000, 2001, and/or 2006 South African Ironman Triathlons	C-allele carriers, *n* = 284	C-allele non-carriers, *n* = 191	Peripheral blood leucocytes	GG *n* (%) = 205 (0.41) CG *n* (%) = 217 (0.43) CC *n* (%) = 82 (0.16)		C, *n* (%) = 381 (0.38) G, *n* (%) = 627 (0.62)		The AQP1 rs1049305 (C > G) variant was associated with running performance. Triathlete carriers of the C-allele completed the 42.2-km run stage faster than triathletes’ non-carriers of the C-allele.			
								Hardy-Weinberg equilibrium *p* = 0.059							
								Genotype by C-allele status				CC or CG: mean = 286 ± 49 min GG: mean = 296 ± 47 min		280–292 min 289–303 min	0.032
								Carriers CC+CG *n* = 299	Non-carriers GG *n* = 20						

The observed allelic frequency distributions within the cases (fast runners) and controls (slow runners) revealed no sex differences (*X*^2^, *p* ≥ 0.05). The sex-pooled allelic frequency distribution revealed significant differences (*X*^2^ = 7.55, *p* = 0.005) between cases (fast runners) and controls (slow runners). In both sexes, within cases and controls, the C-allele was the less frequently observed. The calculated odds ratio = 1.35 and its 95% confidence interval (CI) (1.08–1.67) suggested that the C-allele was more likely (*p* = 0.005) prevalent in the cases (fast runners) than in the controls (slow runners). It is noteworthy that the *AQP1*gene rs1049305 (C > G) is in the 3′ UTR. The 3′ UTR of messenger RNA has been associated with regulating gene expression [34]. The 3′ UTR controls the nuclear export, sub-cellular targeting, and rates of translation and degradation of DNA. The genes controlled by the sequence of the 3′ UTR are generally regulatory proteins, and their irregular expression may have serious effects on humans [35].

### AQP1 Channel and CE Performance

Xu et al. [32], using mice, provided evidence for an association between the Aqp1 channel and CE performance. They tested the hypothesis that the Aqp1 channel plays a physiologically influential role in O_2_ transport, since the Aqp1 channel is present at high levels in erythrocytes and the pulmonary capillary endothelium. They compared voluntary wheel running over a 24-h period in Aqp1-null vs. wild-type mice under conditions of hypoxia (ambient [O_2_] = 16%), normoxia (21%), and hyperoxia (40%). Linear regression analysis of distance run as a function of Aqp1 status and [O_2_] treating [O_2_] categorically referring to 21% O_2_ indicated that the Aqp1 knockout reduced the distance run by 4.7 ± 0.5 km (*p* < 0.001), adjusting for [O_2_]. Compared to 21% O_2_, reducing O_2_ to 16% reduced the distance run by 1.6 ± 0.6 km (*p* = 0.01), while increasing O_2_ to 40% increased the distance run by 1.2 ± 0.6 km (*p* = 0.04), adjusting for Aqp1 status. These findings led to the conclusion that the Aqp1-null mice have a major effect in voluntary exercise tolerance (CE performance), consistent with the hypothesis that Aqp1 plays an important physiological role in O_2_ transport across plasma membranes. It is well accepted that in humans the execution of prolonged exercise (like distance running) depends highly on molecular mechanisms mostly related to the management of O_2_.

### AQP1 Channel and CE Performance Correlates

The present observational study of the AQP1 channel shed further light on the possible role of a molecular mechanism, like that related to the AQP1 channel presence or absence and the acute response to exercise and O_2_ management. In humans, prolonged exercise capacity as that required by long-distance running is highly influenced by VO_2max_, metabolic economy, lactate threshold, temperature regulation, and fatigue resistance. Abundant information indicates the genetics mediate the magnitude of these mechanisms [2]. Of those five factors, the primary determinant of endurance exercise performance is the VO_2max_ [36]. One of the strongest arguments for such contention is that endurance performance and VO_2max_ are strongly and positively associated. New findings [37] arising from a systematic review of 15 studies and meta-analysis indicated that the weighted means for heritability of VO_2max_ absolute values and those adjusted for body weight and for fat-free mass were 0.68 (95% CI 0.59–0.77), 0.56 (95% CI 0.47–0.65), and 0.44 (95% CI 0.13–0.75), respectively. The meta-regression analysis revealed that sex could partially explain the heterogeneity in the VO_2max_ heritability estimates adjusted by body weight. The heritability estimates reported among the studies were statistically significant. Last, for submaximal endurance, phenotypes and endurance performance heritabilities were 0.49 (95% CI 0.33–0.65) and 0.53 (95% CI 0.27–0.78), respectively.

### AQP1 C-Allele Carrier Status and CE Performance

In humans, the second line of support for the hypothesis of an association between the *AQP1* gene and CE performance was shown by Rivera et al. [29] (see Table 1). For a second time, an observational study using a genetic epidemiology model evaluated the association between the DNA sequence variant, rs1049305 (C > G), in the 3′ UTR of the *AQP1* gene and the CE performance-related phenotype. In this occasion, elapsed running time in a 10-km event was compared by *AQP1* C-allele carrier status, e.g., carriers (homozygous for C-allele (CC) and heterozygous for C-allele (CG); *n* = 50) and non-carriers (homozygous for G-allele (GG); *n* = 41). The main findings indicated that *AQP1* C-allele carries ran an average of 13.4% faster (*p* < 0.05) than non-carriers during the 10-km race, which is approximately 16.12 km/h for carriers and 13.9 km/h for non-carriers. There was no difference in training status between the two groups (carriers vs non-carriers of the *AQP1* C-allele). These findings provide further support to the notion that inter-individual variability in CE performance could be partly explained by molecular mechanisms, such as DNA sequence variations. The findings of Rivera et al. [29] provide additional support to those of Martinez et al. [28], suggesting the participation of *AQP1* rs1049305 CC and CG genotype in promoting endurance running performance level.

In humans (see Table 1), a third line of evidence provided further support for the possible role of *AQP*1 genotype in CE performance. This time, the association between CE performance and the rs1049305 (C > G) variant within the 3′ UTR region of the *AQP1* gene was evaluated in South African Caucasian male (*n* = 504) finishers in either the 2000 (*n* = 112), 2001 (*n* = 222), and 2006 (*n* = 170) South African Ironman Triathlons [30]. Their results replicated those of Martinez et al. [28] and Rivera et al. [29] by reporting that the *AQP1* rs1049305 C-variant was associated with the duration of marathon running segment in three Ironman events. Triathletes who carried the C-allele completed the 42.2-km run stage faster (mean 286, *s* = 49 min) than triathletes with the GG genotype (mean 296, *s* = 47 min; *P* = 0.032). That study also contended that their findings and those of Martinez et al. [28] and Rivera et al. [29] are not predictors of endurance performance but are evidence that the *AQP1* rs1049305 C-variant contributes to a physiological state receptive to training and beneficial to endurance (long distance) running performance. Some further argue that the weakness of observing a similar genotype effect on performance in the swim and bike stages likely reflects the differing physiological requirements of these activities [30].

### Expression, In Vitro, and *AQP1* G Allele

One report [38] revealed, that in vitro (see Table 2), a reduced AQP1 expression was associated with the presence of the rs1049305 G-allele. It was postulated that such reduction in expression of AQP1 could be attributed to an increase in the binding affinity of a microRNA-129 precursor to its binding site two base-pairs (bp) away from the rs1049305 [38]. The same study indicated that with liver fibrosis patients, the AQP1 rs1049305 CC genotype was associated with lower serum sodium concentration and lower serum osmolality when compared to patients with a CG or GG genotype. Saunders et al. [30] hypothesized that reductions in the expression of AQP1 in the presence of the G-allele could cause a slower response to changes in osmotic gradient during exercise. That notion substantiated by the Tam and Noakes [4] observation that serum osmolality is physiologically defended during exercise.

**Table 2:** Summary of study evaluating the influence of the rs1049305 (C > G) in the AQP1 gene expression, in vitro

Source (ref no.)	Study design	Purpose	Exclusion criteria	Subjects	DNA source/genotyping method	In vitro study/gene expression	Genotype frequencies	Allele frequencies	Analysis	Main finding	*p* value
38	Prospective study that collected data from gastroenterology and hepatology patients with cirrhosis and ascites	To investigate the distribution of single-nucleotide polymorphisms of AQP1 rs1049305 (C > G) and AQP2: rs3741559 (A > G) and rs467323 (C > T) and to analyze their relationship with dilutional hyponatremia. Also, evaluated the possible influence of the rs1049305 (C > G) in the AQP1 gene expression	Exclusion criteria were as follows: history of clinical signs of heart disease, malignant disease, diabetes insipidus, arterial hypertension, or parenchymal renal failure, treatment with corticosteroids, lithium, cyclooxygenase inhibitors, or other nephrotoxic drugs 30 days prior to the study	*N* = 100, Caucasian (Santander, Spain) cirrhotic patients with ascites	Peripheral blood leucocytes genotyping was performed using the Custom Taqman SNP Genotyping Assays	Luciferase assays in vitro to evaluate influence of rs1049305 (C > G) in gene expression	CC *n* (%) = 15 (0.15) CG *n* (%) = 49 (0.49) GG *n* (%) = 36 (0.36) Hardy-Weinberg equilibrium *p* = 0.80	C *n* (%) = 79 (0.305) G *n* (%) = 121 (0.605)	Luciferase assays were evaluated using a non-parametric Mann–Whitney test (two-tailed)	The plasmid corresponding to the C-allele produced a luciferase activity of about 60% of the vector. The C > G change revealed a further 12% decrease in the luciferase activity	0.039

### Prevalence of AQP1 C Allele

Kenyan and Ethiopian runners have dominated Olympic middle- and long-distance running events since the 1968 games in Mexico City [39]. The population distribution of the *AQP1* gene C-allele might partially explain this phenomenon. A report from the National Center for Biotechnology Information [40] found small variations in the frequency (%) of the *AQP1* C-allele between Europeans (0.30 % ), Asians (0.38 % ), and Caucasians (0.42 %), but a striking prevalence of the *AQP1* C-allele in African Americans (0.86 %) and Sub-Saharans (0.98 %). Others [28] reported that in Hispanics the prevalence of the C-allele was 0.36 % in fast runners (cases) and 0.30 % in slow runners (controls).

### AQP1 Channel Activity Under Hypoxic Exercise

Huang and Wang [31] used a different approach to the study of *AQP1* gene and endurance exercise. They examined the effects of aerobic interval training (AIT) and moderate continuous training (MCT) on osmotic stress-mediated rheological function and AQP1 channel activity of human erythrocytes under hypoxic exercise (HE) stress in humans. Thirty healthy sedentary males were randomly assigned to either the AIT group which performed 3-min intervals at 40% and 80% VO_2max_, *n* = 15, or the MCT group required to perform sustained exercise at 60% VO_2max_, *n* = 15, for 30 min/day, 5 days/week for 6 weeks. Erythrocyte rheological responses to HE (100 W under 12% O_2_ for 30 min) were determined before and after various regimens. The findings revealed that acute HE increased osmotic fragility and decreased deformability of erythrocytes, and depressed erythrocyte AQP1 activity as indicated by increased magnesium chloride (HgCl_2_-) induced instability of erythrocyte membrane under hypotonic conditions. After the 6 weeks of exercise intervention, the AIT group exhibited higher maximal power output and VO_2max_ than the MCT group. Both AIT and MCT diminished the extents of enhanced osmotic fragility, reduced deformability, and AQP1 activity of erythrocytes caused by HE. They concluded that AIT was superior to MCT for enhancing aerobic capacity. Either AIT or MCT effectively alleviated the impairments of erythrocyte rheological characteristics and AQP1 function evoked by HE.

### AQP1 and Body Fluid Loss During Exercise

Along these lines, Rivera et al. [29] and Saunders et al. [30] examined body fluid loss (weight changes) association with the *AQP1* rs1049305 (C > G) variant during endurance runs. Rivera et al. [29] reported that during a 10-km road race, carriers of the *AQP1* rs1049305 C-allele had a greater adjusted body fluid loss (3.7 ± 0.9 kg) than non-carriers (1.5 ± 1.1 kg) (*P* < 0.05). Saunders et al. [30] reported no genotype effect on absolute body weight changes in response to the 42-km running segment of Ironman Triathlons. The latter study argued that the observed opposing findings were attributed to methodological issues [30]. In the Ironman Triathlon study of Saunders et al. [30], before and after absolute values of body weight were used as indicators of body fluid loss. Conversely, Rivera et al. [29] determined body fluid loss from the difference between nude body weight (weight before 10 km − weight after 10 km) with adjustments for fluid intake, respiratory water loss, and urine excretion. Tam and Noakes [4] reviewed the literature pertaining to the controversy of when and why absolute body weight should be adjusted, given practical and scientific endeavors. It is beyond the present review to go further into such controversy.

The exercise-induced body fluid loss differences, by AQP1 genotype, observed by Rivera et al. [29] may also indirectly explain the AQP1 association with running performance. The observation that a high body fluid loss is associated with faster running performance in endurance events is not an isolated event. As found by Saunders et al. [30], triathlete finishers of the 2000, 2001, and 206 South African Ironman Triathlon who lost the most body weight during the whole race had better (faster) finishing times than triathletes who lost less body weight. In addition, others had reported significant inverse correlations between body weight changes because of participation in a 100-km ultramarathon (*n* = 50; *r* = − 0.31; *p* = 0.023). Faster runners lost more body mass compared with slower runners while also drinking more [41].

A relevant finding of this systematic review is that during osmotic stress, such as intense exercise [26, 42], AQP1 facilitates the transfer of water from blood into muscle via rapid trans-epithelial transport [43], assists in body fluid balance in various systems, provides osmotic protection, and serves as a conduit for water reabsorption and thermal control [14, 42]. The AQP1 channel, due to its known biological functions, could promote cellular homeostasis during intense exercise by action on nitric oxide and CO_2_ transport [12], two factors linked to endurance performance and prolonged exercise [6, 29]. Wakayama [44] hypothesized that AQP1 might speed skeletal muscle regeneration because of its role in enhancing intramuscular endothelial function. Athletes with the more active AQP1 gene C-allele might train harder and recover faster [29]. More active AQP1 channels in the skeletal muscle and sweat glands might provide several advantages in endurance athletes. They might promote cooling via increased convective heat transfer and sweat rate [26, 45, 46]. Sugie et al. [6] found that AQP1s in erythrocytes were critical for body water management throughout the body.

### AQP1 Null Individuals

In humans, AQP1 null individuals led normal lives and were entirely unaware of any physical limitations [14]. However, they could not maintain fluid homeostasis when exposed to subacute or chronic fluid overload.

## Discussion

The central finding of the present systematic review was the independent replication of the association between *AQP1* gene sequence variant rs1049305 (C > G) in the 3’ UTR and CE performance [28–30]. Further support to the likelihood that the *AQP1* gene and channel are related to CE performance was substantiated by peer-reviewed publications [6, 14, 17, 32, 38, 40, 42–44]. Successful performance in endurance running is a complex, multi-factorial trait, also known as phenotype. A phenotype (e.g., endurance running performance) is the ultimate physical expression of DNA and is caused by the interaction of many proteins evolving from DNA. Such processes can get complicated. Thousands of genes create thousands of different proteins that later interact in complex ways, influenced by diet, training, and environmental/ambient conditions. Performance is dictated by the interaction between environment and the intrinsic genetic factors arising from our individual DNA. Basically, each gene can produce a single protein, which can take several forms. The *AQP1* gene rs1049305 (C > G) have three forms: CC, CG, and GG. The present systematic review findings favor the hypothesis that CC and CG forms are apparently positive contributors to the complex physiological state receptive to training and beneficial to CE (long distance) running performance level, while other form of the gene (GG) was seemingly less functional or poorly associated [28–30].

## Limitations

The main limitation of the present review was the limited number of studies with “adequate sample sizes” in various racial and ethnic groups precluding to perform proper in-depth statistical analysis.

## Conclusions

The *AQP1 *gene rs1049305 C-allele appears to provide for the enhancement of homeostatic mechanisms, yet to be understood, that are auxiliary in achieving an advantage during CE running exercise. The C-allele seems to allow for a greater fluid loss and fluid intake in fast versus slow runners. Perhaps, the *AQP1* gene rs1049305 C-allele and the AQP1 channel might facilitate more intense training, faster recovery, and enhanced temperature regulation. Greater AQP1 [14] activity in erythrocytes with the rs1049305 C-variant allows greater fluid distribution throughout the body, while promoting cellular homeostasis and CO_2_ transport. AQP1s are underappreciated structures that play vital roles in cellular homeostasis at rest and during CE running exercise.

The *AQP1* gene and channel seems to support homeostatic mechanisms, yet to be totally understood, that are auxiliary in achieving an advantage during endurance running exercise. AQP1 functions are vital during exercise and have a profound influence on endurance running performance.

## Data Availability

This study examined published results in each cited reference. All references are readily available in public search engines.

## Abbreviations

3′ UTR: 3′ Untranslated region
AIT: Aerobic interval training
AQP1: Aquaporin-1 channel
*AQP1*: Aquaporin-1 gene
AQPs: Aquaporins
CC: Homozygous for C-allele
CE: Cardiorespiratory endurance
CG: Heterozygous for C-allele
cGMP: Cyclic guanosine monophosphate
CI: Confidence interval
CINAHL: Cumulative Index of Nursing and Allied Health Literature
CO_2_: Carbon dioxide
DNA: Deoxyribonucleic acid
EMBASE: Excerpta Medica database
GG: Homozygous for G-allele
HE: Hypoxic exercise stress
HgCl_2_-: Magnesium chloride
HWE: Hardy-Weinberg equilibrium
MCT: Moderate continuous training
O_2_: Oxygen
PCR: Polymerase chain reaction
rs: Reference single nucleotide polymorphism cluster identification number
SNPs: Single nucleotide polymorphisms
UV: Ultraviolet
VO_2max_: Maximal oxygen consumption
